# Thermodynamic assessment of evaporation during molten steel testing onboard the International Space Station

**DOI:** 10.1038/s41526-024-00416-1

**Published:** 2024-07-19

**Authors:** Jannatun Nawer, Brian Stanford, Matthias Kolbe, Stephan Schneider, Stéphane Gossé, Rainer K. Wunderlich, Markus Mohr, Aurelio Borzì, Antonia Neels, Douglas M. Matson

**Affiliations:** 1 Department of Mechanical Engineering, Tufts University, Medford, MA USA; 2 Institut für Materialphysik im Weltraum, Deutsches Zentrum für Luft- und Raumfahrt (DLR), Köln, Germany; 3 Université Paris-Saclay, CEA, Service de Corrosion et du Comportement des Matériaux, Gif Sur-Yvette, France; 4 Institute of Functional Nanosystems, Ulm University, Ulm, Germany; 5 Institute of Quantum Technologies, German Aerospace Center (DLR), Wilhelm-Runge-Straße, Ulm, Germany; 6 Center for X-ray Analytics, Empa Swiss Federated Laboratories for Materials Science and Technology, Dübendorf, Switzerland

**Keywords:** Theory and computation, Thermodynamics

## Abstract

Evaporation control is a critical facility resource during solidification experiments that limits processing time and must be tracked to ensure facility health. A thermodynamic analysis was performed on a ternary FeCrNi sample processed onboard the International Space Station (ISS) using ESA Electromagnetic Levitation (EML) facility in a microgravity environment. A non-ideal solution-based mathematical model was applied for the overall sample mass loss prediction during this study. The overall sample mass loss prediction is consistent with the post-flight mass loss measurements. The species-specific findings from this study were validated using post-mission SEM-EDX surface evaluations by three different facilities. The bulk composition prediction was validated using SEM-EDX and wet chemical analysis. The non-ideal solution model was then applied to predict the composition of the dust generated during EML testing. The thicknesses of the deposited layer on the EML coil at various locations were also calculated using the geometry of the facility and results were validated with near-real-time dust layer predictions from toxicity tracking software developed by the German Space Center (DLR) Microgravity User Support Center (MUSC).

## Introduction

Design optimization of additive manufacturing^1^ greatly depends on the control of unwanted evaporation and the knowledge of potential changes in concentration of alloying elements. Dynamic tracking of these quantities is crucial to support modeling and simulation of these processes^2–4^. The generation of aerosol dust through evaporation is an inherent aspect of steel smelting^5,6^. This dust may be comprised of minuscule particles that may be toxic to humans thus making it imperative to minimize dust production for the sake of resource conservation, safeguarding facility health, and ensuring operator safety. During space testing, monitoring dynamic mass loss is essential not only for maintaining a deposition below the specific astronaut toxicological exposure safety limits but also to ensure an accurate and precise measurements of the thermophysical properties of the material such as density, thermal expansion coefficient, viscosity and surface tension^7,8^.

Vaporization kinetic models based on Langmuir’s equation have been widely applied in modeling various material processing methods^9–11^. Butorina^12^ used a mathematical model to prove that the dominant mechanism of dust generation above a metal melt is evaporation compared to the dispersion in the absence of any active combustion of carbon from the melt with oxygen injection. This work also predicted the rate of dust formation and particle size using an electric furnace with no prediction of the species-specific composition of the generated dust after condensation. In the present work, the species-specific mass loss due to evaporation was predicted by using a non-ideal solution-based Langmuir model which was validated by both surface and bulk analysis. The prediction of the generated dust was also confirmed by doing a surface analysis of the sample holder during this study.

Previous thermodynamic assessment using an ideal solution model based on Langmuir’s equation was developed^13^ with limited success to predict the rate of evaporation for multi-element alloys processed using NASA Marshall Space Flight Center (MSFC)’s ground-based Electrostatic levitation (ESL) facility under vacuum conditions and ESA International Space Station (ISS) Electromagnetic Levitation (EML) facility. This model was later modified to consider non-ideal activity of individual alloy constituents and was successfully implemented to predict mass loss due to evaporation for two Ni-based superalloys CMSX-10 and MC2 using ISS-EML^14^ under Argon (Ar) gas atmosphere; Ni-based CMSX-4 Plus® (SLS) using ground-based MSFC ESL under vacuum condition and Japanese Aerospace Agency’s space-based ISS-ELF (Electrostatic Levitation Furnace) facility^15^ under Ar atmosphere. In this study, the non-ideal solution-based model was extended to predict volatile mass loss and dust generated during testing of ternary Fe-Cr-Ni stainless steel alloys conducted as a part of a series of Batch-1 ISS-EML experiments^16,17^.

**Fig. 1:**
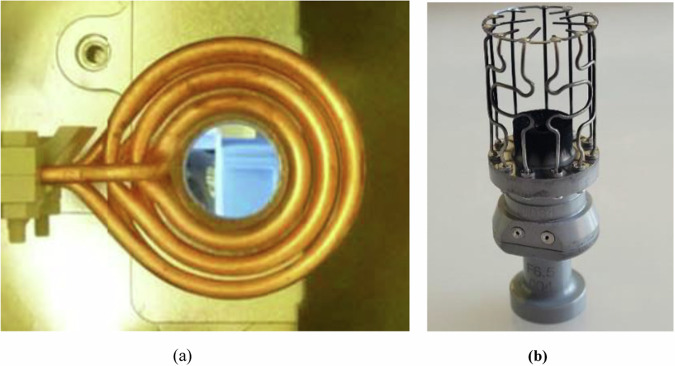
ISS-EML facility images. **a** EML inner coil and **b** sample holder for Batch-1 FeCrNi Sample #09.

A typical EML test is conducted either under 2.35 ± 0.17 × 10^−7^ mbar high-vacuum condition or under an inert shielding gas atmosphere 356 ± 2 mbar to limit evaporation. At low pressures, evaporation of atoms from the heated sample surface form aerosol particles which may agglomerate over time inside the processing chamber. The aerosols can form a fluffy layered structure on exposed surfaces such as the inner coil of EML facility and the sample cage holder as shown in Fig. 1. These structures were investigated during this study to determine the elemental composition of the deposit generated. In a gas environment, the evaporation flux is reduced near the droplet surface because of the presence of any adjacent molecules; this is called the vapor-shielding effect. This can be achieved by the use of an inert gas which significantly decreases the evaporation rate compared to vacuum. This phenomenon is modeled using a shielding factor which represents the percentage reduction in flux into inert gas as compared to the flux if the inert gas was not present – as is the case for vacuum testing^18^.

The purpose of this paper is to provide an opportunity to validate the analytical prediction of the deposit generated due to evaporation to measurements conducted during surface analysis of the sample holder. This study explores four methods to check the model: (1) total bulk mass loss, (2) species-specific mass loss, (3) bulk concentration shift, and (4) evaporation deposit composition prediction. In order to validate the analytical model, all four methods need to agree. Total bulk mass loss prediction was validated using post-processing sample mass, species-specific mass loss was validated using surface analysis using Scanning Electron Microscopy (SEM), bulk concentration shift was determined from the SEM analysis on both sample surface and in the bulk, and evaporation deposit composition prediction validated using the SEM on the sample surface holder. The dust deposition was calculated using facility geometry and validated using “Tox Tracker” software by the German Space Center (DLR) Microgravity User Support Center (MUSC) which monitors real time toxicity level due to dust generation during an EML test.

## Results and discussion

A total of 54 melt cycles were performed for a FeCr_21_Ni_19_ (at.%) sample during Batch-1 processing of ISS-EML: 3 cycles in vacuum, 25 melt cycles in Helium (He) gas, and 26 melt cycles in Argon (Ar) gas atmosphere. The results from the mathematical modeling are as follows:

### Estimation of mass loss due to evaporation

**Fig. 2:**
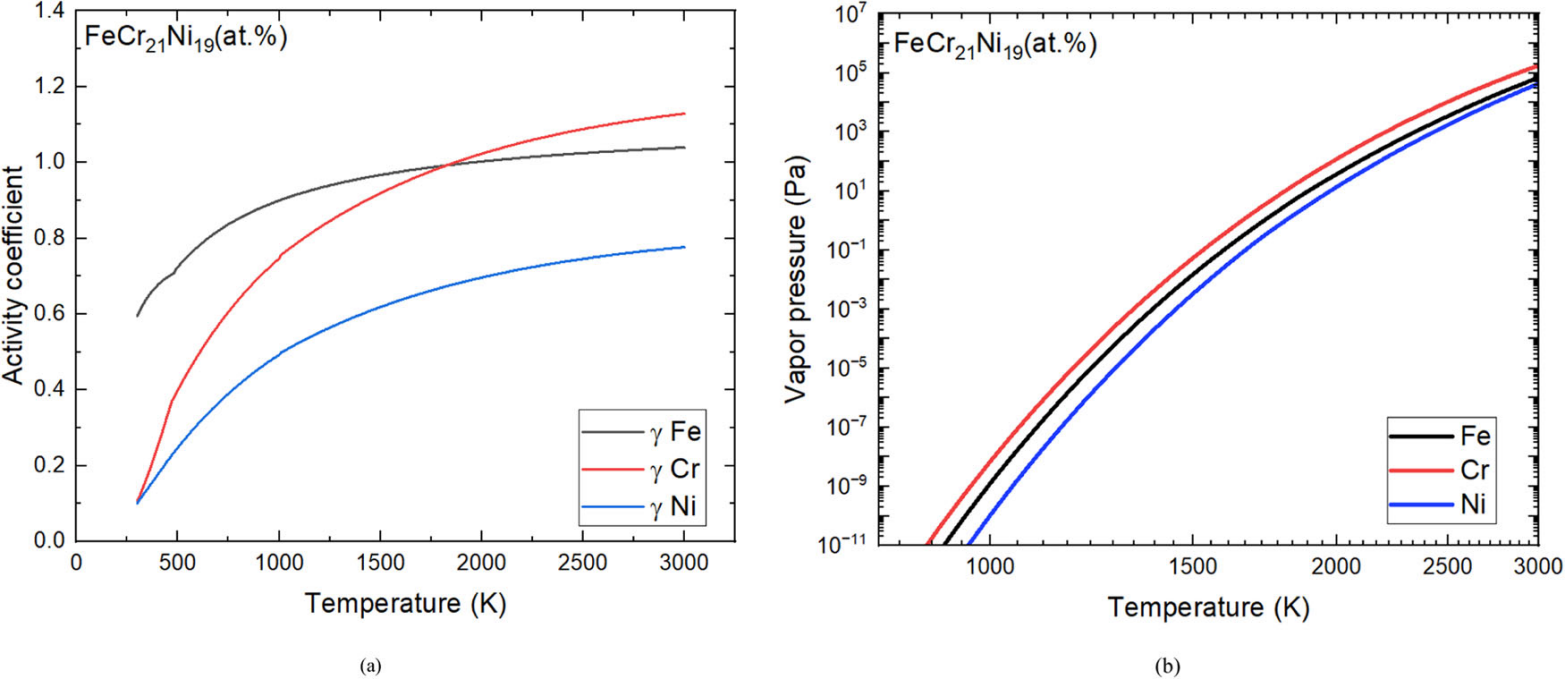
Thermo-Calc results for FeCrNi components as a function of temperature. **a** Activity coefficients and **b** non-ideal solution vapor pressure. Black lines represent Fe, Red lines represent Cr and Blue line represents Ni.

The chemical activity for the three individual alloying elements (Fe, Cr, and Ni) were calculated using the Thermo-Calc for temperature ranges corresponding to solid, liquid, and mushy zone mixture. This software calculates the thermodynamic properties of complex chemical systems from databases containing the free enthalpy functions of all phases: solids, liquids and gases. These databases, many of which are available as open science, are developed from experimental data and atomistic calculations. The chemical activities for this study were calculated using the Thermo-Calc TCFE8 database which are derived from the free enthalpy of the system and are established by minimizing the overall energy of the phases at equilibrium. Activity coefficients were then calculated to account for the non-ideal solution behavior of the steel ternary analog alloy as shown in Fig. 2a. Both Cr and Fe exhibit ideal solution behavior above 1900 K. Activity coefficient of Ni is slightly lower than that of the other elements. Non-ideal solution vapor pressures for each element were calculated using the activity coefficients and vapor pressure literature data from Alcock^19^. Cr has the highest vapor pressure and Ni has the lowest vapor pressure in this alloy system as shown in Fig. 2b. This indicates that Cr is more prone to evaporation compared to Fe and Ni during the superheating phase.

Based on the knowledge of activity-corrected vapor pressure, the evaporation model was applied for a discreet time interval of 0.01 s for each constituent throughout the 54 thermal cycles in the order of experiment sequence as set by the facility pyrometer data acquisition rate. Table 1 shows the applied average shielding factors used for the model and relevant mass loss prediction from the non-ideal solution model for different atmospheres in chronological order. A majority of the mass is predicted to be lost within the first 3 cycles run in vacuum condition without any gas shielding present. Application of Argon and Helium gases significantly reduced the mass loss prediction per cycle with an average of 0.03 mg in Argon and 0.02 mg in Helium atmosphere. The total mass loss predicted by the analytical model is 8.41 mg. The vapor pressures used for these measurements have an accuracy of ±5% as reported by Alcock^19^. The density has a reported accuracy of ±15%^20^. The one color high-precision pyrometer used for the temperature detection has an accuracy of ±5 K (resolution of <0.1 K above 873 K)^14^. Fromm^18^ reports an uncertainty of about a factor of three on the shielding factor reported in Table 1. Other sources of error include the computational error in quantifying the element-specific activity coefficients which is hard to establish due to numerous phase diagram data and thermodynamic quantities used to optimize the alloy system. Given the uncertainties present in the modeling parameter, the precited mass loss is in excellent agreement with the actual mass loss.

**Table 1:** Overview of predictions from the analytical model

Atmosphere	No. of cycles	Shielding factor	Predicted mass loss (mg)		
			Fe	Cr	Ni
Vacuum	3	-	3.14	3.63	0.33
Helium	25	$\frac{1}{171}$	0.17	0.20	0.01
Argon	26	$\frac{1}{427}$	0.40	0.45	0.04
**Total**	**54**	**-**	**3.72**	**4.29**	**0.39**

### Compositional elemental analysis

The result from the analytical prediction was validated using the post-processed bulk and surface analysis using SEM-Energy Dispersive X-ray (EDX) Spectroscopy and Wet chemical analysis using Direct Current Plasma Emission Spectroscopy (DCPES) as listed in Table 2. An unpaired t-test was conducted for all three elements between three facility surface results which concludes that the datasets are not statistically different (*p* > 0.05). It is significant that the post processing measurements at independent laboratories agree. These results also indicate an enrichment of Cr on the surface. This is unexpected as Cr is the most volatile species and we would expect its concentration to be decreased following processing. The anticipated decrease is predicted accurately during modeling and validated using the post-processing analytical evaluation. Post-test wet chemical analyses of the bulk sample from Table 2 shows that Fe and Cr are depleted by 2.2% and 0.3% respectively and Ni is enriched by 7.2%. Based on *p* values observed from a two-tailed unpaired *t* test, it is concluded that the surface segregation is statistically significant for all elements (*p* < 0.05) as shown in Table 3. Hence, the analytical predictions were calculated by accounting for surface segregation which was less than 0.1% as compared to assuming bulk concentration. For all three constituents, analytical predictions are within 10% of the composition observed from the EDX analysis at Uni-Ulm and at Empa and within 2.6% of the wet chemical DCPES analysis.

**Table 2:** Post-processing surface and bulk analysis along with model prediction

Element (wt%)	Surface			Bulk		Model prediction
	Ulm-EDX	Empa-EDX	DLR-EDX	DLR-EDX	DCPES	
Fe	58.80 ± 0.30	59.7 ± 0.4	58.54 ± 0.89	59.84 ± 0.15	60.60 ± 1.2	60.41
Cr	21.00 ± 0.61	20.4 ± 0.1	21.57 ± 0.57	21.62 ± 0.10	19.80 ± 0.40	19.56
Ni	21.20 ± 0.16	19.9 ± 0.3	19.88 ± 1.11	18.53 ± 0.11	19.50 ± 0.40	20.01

**Table 3:** Two-tailed unpaired t-test results for surface vs bulk analysis from DLR-EDX

Variable	DOF	Standard error	*t* value	*P* value
Fe	16	0.30	4.32	0.0005
Cr	16	0.19	155.20	0.0001
Ni	16	0.372	3.6309	0.0022

### Deposited layer composition-SEM evaluation

**Fig. 3:**
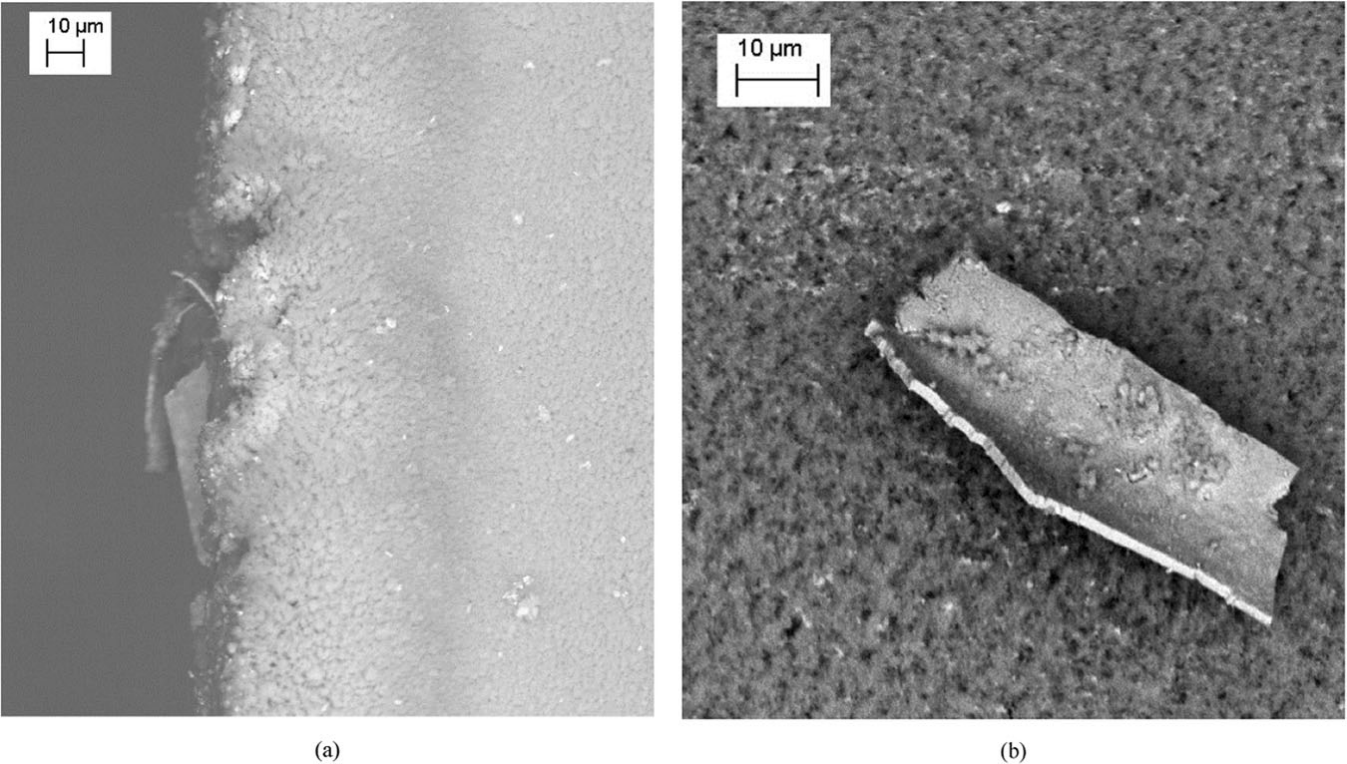
Images from SEM tests conducted during the post-processing. **a** An impact region on the pedestal and **b** an impact chip from the sample cage wire.

SEM-EDX evaluation of the sample surface and ISS-EML sample holder cage were performed at DLR as shown in Fig. 3. Figure 3a shows SEM images of an impact region where the sample collided with the pedestal along with a fragment shown in Fig. 3b which was detached from the cage wires. The SEM results conducted within these regions are listed in Table 4 along with the predicted deposit composition from the analytical model. The predicted composition for Ni from the non-ideal solution model is in good agreement with the SEM-EDX observation. As expected, the deposition layer is significantly enriched in the more volatile Cr and significantly depleted in Ni.

**Table 4:** Validation of evaporated material composition model

Element (wt%)	Chip observation EDX (DLR)	Predicted deposit calculation
Fe	38.2	44.2
Cr	57.5	51.0
Ni	4.3	4.6

### Deposited layer thickness - cage geometry

The ideal layer thickness during the EML testing can be measured based on the assumption of a spherical shell located at a known distance from the center of the sample. The total loss of mass is 8.9 mg which is known from the pre-test and post-test mass measurements. Assuming the deposited layer is 100% dense, using room temperature density, this mass corresponds to a volume of 1.128 mm^3^. The deposited layer thickness can be estimated at any radius and these predictions are listed in Table 5 for key facility locations where deposit may accumulate.

**Table 5:** Layer thickness predictions for key facility locations

	Radius (mm)	Layer thickness (nm)
Center to cage wire	7.25	1707
Center to pedestal base	6.85	1912
Center to inner coil	9.89	**918**
MUSC coil prediction	- -	**1032**

The approximate layer thickness can be estimated using Fig. 3 as the layer edges are clearly evident in conjunction with the associated micrograph scale. The observed layer thickness is in the order of 1140 nm with unknown layer density. The mass loss prediction for coil deposition at 100% packing density is 918 nm which shows excellent agreement with the prediction of 1032 nm from the MUSC Tox Tracker tool.

## Methods

### Materials

A FeCr_21_Ni_19_ (at.%) sample with a pre-test mass of 1.1509 g and an average diameter of 6.456 ± 0.050 mm was tested in microgravity during this study. This sample was made from high purity Fe, Cr and Ni using a suction mold technique in a water-cooled copper mold attached to an arc melter at Ulm University. The post-test sample mass was 1.142 g with a total mass loss of 8.9 mg. Key material properties used for the mathematical modeling are listed in Table 6.

**Table 6:** Fe-Cr-Ni Material properties^21^

Properties	Value
T_s_ (K)	1706
T_l_ (K)	1715
*ρ*_*s*_ at T_s_ (kg·m^−3^)	7900
*ρ*_*l*_ at T_l_ (kg·m^−3^)	6991

### Experimental facility

**Fig. 4:**
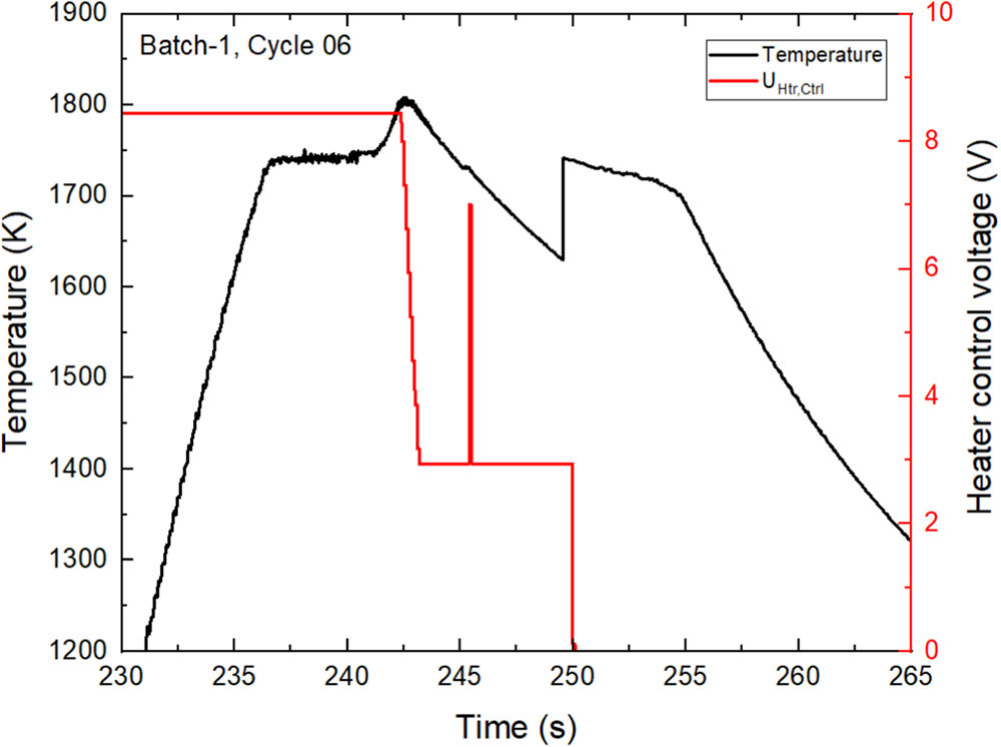
A typical thermal profile of FeCrNi processed on the ISS-EML facility during Batch-1 Sample #09. Black line represents temperature recorded from the pyrometer and red line shows heater control voltage. This melt cycle was run in a He gas atmosphere.

The experiments for this study were conducted using the ESA ISS-EML facility^21,22^ developed by Airbus Defense and Space^23,24^. This powerful containerless technique simultaneously allows researchers to levitate and thermally condition molten metal droplets providing the advantage of processing samples without adverse impacts related to sedimentation, chemical reactions, or heterogeneous nucleation on crucible walls. The extended period of microgravity environment also enables the opportunity to conduct long duration experiments in the undercooled regime under a wide range of controlled flow conditions^25–27^. A typical thermal profile from this facility during FeCrNi experimentation is shown in Fig. 4. The black line represents the temperature profile with time as measured by a pyrometer with an acquisition rate of 100 Hz. The red line represents the heater control voltage setting that is used to control cooling rate and internal convection within the sample. Note that in the cycle shown in Fig. 4, during the cooling phase from (242 to 250) s a heater control voltage setting was applied to intentionally induce internal convection to investigate the influence of stirring on solidification behavior. Additionally, a heater pulse was applied at around a time of 246 s to excite surface oscillations to conduct thermophysical property evaluations in the undercooled state.

### Metallographic facilities

Post-test compositional surface analysis of the spherical sample was conducted in three different experimental facilities. The first set of analyses was conducted at Ulm University, Ulm, Germany using a Zeiss Leo 1550FE equipped with an EDX spectrometer from Oxford Instruments. Special care was taken to perform the measurement on a small area on the top of the sphere, in order to avoid possible systematic measurement errors due to the curvature of the surface.

The second set of analyses was conducted at the Center for X-ray Analytics, Empa Materials Science and Technology, Dübendorf, Switzerland. 2D X-ray diffraction analysis (2D-WAXD; STOE IPDS-II, 0.71073 Mo Kα radiation source) was carried out at the sample surface to confirm the cubic structure of the FeCrNi alloy with the space group Fm-3m and *a* = 3.56 Å. EDX was conducted at different positions of the sphere surface. Minor C and Al contamination was present on the sphere surface and was not been considered in the quantitative analysis. A Zeiss Gemini SEM 460 was used for high-resolution imaging and high-efficiency analytics, namely elemental analysis using an Oxford EDS system (Ulitm Max 170).

The third set of analyses was conducted at the Institut für Materialphysik im Weltraum, Deutsches Zentrum für Luft- und Raumfahrt (DLR) in Köln, Germany using an Zeiss SEM LEO 1530 VP. The EDX-detector is a liquid nitrogen-cooled Si(Li) detector operated under INCA software by Oxford Instruments. This facility was used to conduct surface analysis on the post-processed sample and the sample cage as well as the impact wire chip.

Direct Current Plasma Emission Spectroscopy (DCPES)- ASTM D4190, E1097 was conducted by Luvak Inc, Boylston, MA, USA for wet chemical analysis using a Beckman SpectraSpan IV. The sample was first dissolved in an acid mixture and then analyzed using the DCPES. Argon plasma provided the energy to temporarily excite electrons to the elements’ outer shells. The electrons return to their original shells emitting energy as light at specific wavelengths. Photomultiplier tubes detect the emission intensities at the specified wavelengths for comparison to the emissions of calibration standards created from certified reference materials. Solution standards with known concentrations of the elements to be analyzed were used to establish calibration data for each analysis. Calibration was verified through analysis of certified reference materials. The instrument precision for this method is ~2%.

### Evaporation analysis—analytical approach

Analytical mass loss prediction for the FeCrNi system was performed on a model previously developed by the author^13–15^ based on Langmuir’s equation^28^. The total mass loss ($$v$$) from a surface of an alloy due to evaporation can be measured using:where $$R$$ is the gas constant, $$A$$ is the sample surface area and $$\alpha$$ is correction factor. For each relevant species *i*, *c* is surface concentration, $$\gamma$$ is activity coefficient, $${P}_{v}$$ is vapor pressure and $$M$$ is molecular weight at a given instantaneous temperature ($$T$$) for a discreet time interval $$\triangle t$$. Temperature ($$T$$) was obtained from the pyrometer measurements ($${T}_{P}$$) during each test which was corrected using the liquids temperature ($${T}_{L}$$) and observed liquidus temperature ($${T}_{{PL}})$$ for any emissivity shift due to oxidation^29^ using:

1 $$v=\mathop{\sum }\limits_{0}^{t}\mathop{\sum }\limits_{i=1}^{m}\frac{{\gamma }_{i}{c}_{i}{P}_{v,i}\alpha A}{\sqrt{2\pi {M}_{i}{RT}}}\Delta t$$

2 $$T={\left(\frac{1}{{T}_{P}}+\frac{1}{{T}_{L}}-\frac{1}{{T}_{{PL}}}\right)}^{-1}$$

The changes in surface area were dynamically monitored through edge detection^30^. Vapor pressure, ${P}_{v,i}$ was calculated using the four-termed polynomial expression based on experimental measurements as cataloged by Alcock^19^:

3 $$\log p\,({atm})=A+B\,{\cdot}\,{T}^{-1}+C\,{\cdot}\,\log T+D\,{\cdot}\,T\,{\cdot}\,{10}^{-3}$$

Correction factor $\alpha$ represents the shielding factor which is also known as the deviation in the rate of mass evaporation in an inert gas environment from the ideal evaporation in a vacuum environment ($v/{v}_{0}$). This shielding factor was applied to the metal evaporation rate which were previously determined by Fromm^18^ using:

4 $${{\alpha }_{{Ar}}=[v/{v}_{0}]}_{{Ar}}=\frac{1}{1+K{P}_{G}^{n}}$$

where, ${v}_{0}$ is the mass loss in vacuum, ${P}_{G}$ is inert gas pressure, $K$ and $n$ are two empirical constants. For both Ar and He inert gases, $K$ = 0.012 and $n=1.0$ were used as provided by Fromm^18^. For a vacuum environment, ${P}_{G}=0$ such that evaluation of Eq. 4 results in no shielding. Shielding effect with He is less effective than Ar and $\alpha$ for He gas atmospheres was measured using:

5 $${\alpha }_{{He}}=\frac{{[v/{v}_{0}]}_{{He}}}{{[v/{v}_{0}]}_{{Ar}}}=\frac{\sqrt{1+{M}_{i}/{M}_{{He}}}}{\sqrt{1+{M}_{i}/{M}_{{Ar}}}}$$

Under an Argon gas atmosphere of 350 millibar, the shielding factor becomes ${\alpha}=$ 1/427 while under Helium gas atmosphere the shielding factor is corrected to a value of $\alpha =$1/17 1 using Eq. 5.

This non-ideal solution approach emphasizes thermodynamic prediction of non-ideal solution behavior of each species in solution in conjunction with empirical predictions of the variation of vapor pressure with temperature. It does not address validation of ground-based experimental test results but rather emphasizes tracking of dynamic composition shifts for volatile species.

### Evaporation analysis—empirical approach using MUSC Tox Tracker

The results from non-ideal solution were validated using the Tox Tracker by DLR-MUSC. This is a software-based simulation tool primarily used for ISS-EML experiment analysis. After each space experiment, the Tox Tracker tool is used to track facility health—the potential for deposited material on the coil—and the accumulation of specific toxic species—to ensure astronaut safety—based on global evaporation from the sample as a function of the sample thermal profile. Ground-based global mass evaporation is measured pre-mission and element specific losses are then predicted assuming that individual species exhibit ideal behavior in solution. The Tox Tracker uses the near-real time temperature data from the pyrometer after each melt cycle to calculate for each measured temperature value the evaporated mass. The total evaporated mass is distributed to each element assuming an ideal solution model using the initial concentration of the element weighed with the vapor pressure. The Tox Tracker evaluation is based on real temperature tracking, experimentally verified global evaporation, and extrapolation of results to specific chemical species. This approach does not take into account changes in concentration over time for specific species or non-ideal solution behavior but rather emphasizes prediction of overall mass loss based on experimental validation.

## Data Availability

Data are available upon request from the German Space Agency (DLR).

## Code availability

Code for the mathematical model will be available upon request from the corresponding author.
